# Texas State Medical Association to Meet in Austin April 26, 27, 28 and 29, 1904

**Published:** 1904-03

**Authors:** Jno. T. Moore

**Affiliations:** Secretary Texas State Medical Association; Galveston, Texas


### Texas State Medical Association to Meet in Austin April 26, 27, 28 and 29, 1904.

GALVESTON, TEXAS, February 19, 1904.

*To the Chairmen and Secretaries of Sections:*

As Chairman or Secretary of a Section to present papers to the Texas State Medical Association, I wish to call your attention to Section 4, Chapter 3, page 5, and also to Section 5 of the same Chapter of the Constitution and By-Laws for the State Society.

This provides that papers shall be read within twenty minutes. This will be strictly adhered to, and papers that require more time than this should be read in synopsis. Please urge all persons presenting papers to do this.

Advise all persons presenting papers to have them clearly typewritten, on one side of the paper, and ready to hand to the Secretary as soon as it is read.

Everything points to a large and enthusiastic meeting, and we desire to make the papers the best in the history of the Association.

All titles for papers must be in my hands not later than April 1st. I am,

Fraternally yours,

JNO. T. MOORE,

Secretary Texas State Medical Association.

---

GALVESTON, TEXAS, February 19, 1904.

*To the Councilors, Texas State Medical Association:*

I take this opportunity of calling your attention to the necessity of completing the organization work at as early a date as possible.

You, no doubt, know that unorganized counties have no representation in the State Society, and it is greatly desired that we have a representative from every county that has physicians enough to organize. I might say further that physicians in these unorganized counties can not take part in the meeting or discussion of the State Society. This would work a great hardship upon the men residing in such counties.

The State Constitution and By-Laws provides that the component county societies in each district (Councilor District) be organized in a District Society, and that the Vice-Presidents of the State Society are to be selected from the Presidents of the District Societies.

I would like to urge each of the Councilors to take steps to organize their Districts at once, or at least before the close of March.

The House of Delegates will meet April 25th, at 2 o'clock p. m., this being the day preceding the meeting of the annual session of the Association. The County Societies should be informed of this by you, and they should take the necessary steps to have their Delegate elected and present at that date. The certificate of the Delegate should be forwarded to the Secretary of the State Society as soon as he is elected.

The Fifth, or Western District, has set a splendid example of what can be done in the organization of District Societies. At their organization at San Antonio, ninety members sat down to the banquet table together.

It is hoped that each of the Districts will be able to complete as good a society before the session at Austin, April 26, 1904.

The meeting at Austin promises to be the best in the history of the State Association. I am,

Cordially and fraternally yours,

JNO. T. MOORE,

Secretary Texas State Medical Association.

